# Compile- and run-time approaches for the selection of efficient data structures for dynamic graph analysis

**DOI:** 10.1007/s41109-016-0011-2

**Published:** 2016-09-05

**Authors:** Benjamin Schiller, Clemens Deusser, Jeronimo Castrillon, Thorsten Strufe

**Affiliations:** 1 Privacy and Data Security, Department of Computer Science, TU Dresden, Nöthnitzer Straße 46, Dresden, 01187 Germany; 2 Chair for Compiler Construction, Department of Computer Science, TU Dresden, Georg-Schumann-Straße 7A, Dresden, 01187 Germany

**Keywords:** Dynamic graph analysis, Data structures, Performance, Measurement study, Compile-time optimization

## Abstract

Graphs are used to model a wide range of systems from different disciplines including social network analysis, biology, and big data processing. When analyzing these constantly changing dynamic graphs at a high frequency, performance is the main concern. Depending on the graph size and structure, update frequency, and read accesses of the analysis, the use of different data structures can yield great performance variations. Even for expert programmers, it is not always obvious, which data structure is the best choice for a given scenario.

In previous work, we presented an approach for handling the selection of the most efficient data structures automatically using a compile-time approach well-suited for constant workloads.

We extend this work with a measurement study of seven data structures and use the results to fit actual cost estimation functions. In addition, we evaluate our approach for the computations of seven different graph metrics. In analyses of real-world dynamic graphs with a constant workload, our approach achieves a speedup of up to 5.4× compared to basic data structure configurations.

Such a compile-time based approach cannot yield optimal results when the behavior of the system changes later and the workload becomes non-constant. To close this gap we present a run-time approach which provides live profiling and facilitates automatic exchanges of data structures during execution. We analyze the performance of this approach using an artificial, non-constant workload where our approach achieves speedups of up to 7.3× compared to basic configurations.

## Introduction

There is an emerging application domain that deals with the analysis of dynamic graphs. They serve to model dynamic systems across different disciplines, such as biological (Candau et al. 1982; Marti 2000), transportation (Chabini 1998), computer (Gonçalves 2012), and social networks (Braha 2009; Kossinets 2006; Mucha 2010). The analysis of such dynamic graphs is challenging and its complexity arises from the frequent changes to their topologies and properties rather than their size alone. Due to a proliferation of applications and the ever increasing size of dynamic systems, performance has quickly become a major concern (Ediger 2010, 2012; Madduri and Bader 2009).

**Fig. 1:**
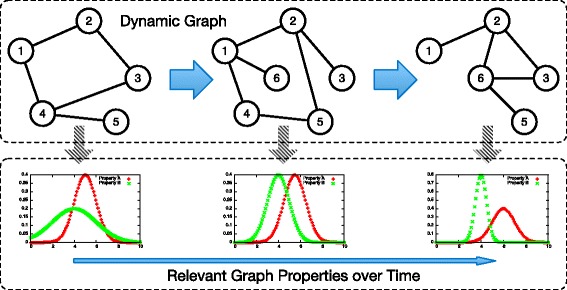
General application scenario of dynamic graph analysis

The general application pattern of dynamic graph analysis consists of a sequence of graph modifications followed by a computation of metrics (cf. Fig. 1). Several metrics investigate local properties such as the clustering coefficient and assortativity. Other metrics determine global properties like degree distribution, all-pairs shortest paths, and connected components. Each metric has a different interpretation depending on the application domain. As an example, a high betweenness centrality identifies users with high influence in social networking and potential communication bottlenecks in computer networks. Such an analysis serves to better understand the states of a system and improve its design in a variety of applications (Ambedkar et al. 2015; Trequattrini et al. 2015; Zhao et al. 2015). The analysis of the states of a dynamic graph can be implemented using snapshot- or stream-based approaches (Ediger et al. 2010). We use snapshot-based algorithms in the following since the problem of modifying and accessing the in-memory representation of a dynamic graph is the same for both.

For performance reasons, dynamic graph analysis is implemented on an in-memory graph representation (Ediger et al. 2010; 2012). There are well understood representations of graphs, such as adjacency lists and matrices, on which algorithms, data structures, and complexity analyses have been studied extensively. For practical applications, however, it remains challenging to find the best suited match of algorithms and data structures as the result often depends on the combination of a number of factors. In the case of dynamic graphs this includes graph size and structure, frequency of updates to its topology, and access patterns of the metric computation. Different graph representations result in high performance deviations but are challenging for programmers to predict (Hunt and John 2011; Shirazi 2003).

There exist many frameworks for the efficient analysis of static graphs (Bader et al. 2008; Batagelj et al. 1998; Malewicz et al. 2010). While they are all built for efficient analysis, the graph representation is fixed and selected by the developers. Many graph databases have been developed to represent graph over time (McColl et al. 2009). While they allow for complex queries of the graph over time and the storage of additional properties, they are neither suited for a large number of updates nor the efficient computation of topological graph properties for specific states (Ciglan et al. 2012). A lot of work has been done to develop compact representations of graphs. These approaches do not focus on runtime efficiency but on obtaining a small memory footprint (Blandford and et al. 2004). They often are not even applicable to arbitrary graphs as they are developed for separable or sparse graphs (Blandford et al. 2003; Sun et al. 2007). Special graph representations for dynamic graphs have also been developed. Their underlying data structures are tuned for memory (Madduri and Bader 2009) or runtime efficiency (Bader et al. 2009; Ediger et al. 2012; Macko 2014) but cannot be adapted to different scenarios.

Many approaches have been developed for profiling programs to facilitate their subsequent optimization. Frameworks like *Pin* (Luk et al. 2005) or *JFluid* (Dmitriev 2004) allow the instrumentation of existing programs to collect statistics about CPU usage, memory consumption, or call frequencies of code fragments. In addition to this instrumentation, *Brainy* (Jung et al. 2011) enables the optimization of the data structures used by a program. Based on benchmarks of available data structures, the approach uses machine learning to generate rules like, e.g., *if operation o is called more than k times use data structure d*. After the analysis of a complete execution of the program, data structures are exchanged based on these general rules. This approach is not applicable to the problem of dynamic graph analysis because the generated rules are generalized for all data types and do not take into account the specific runtime properties of handling vertices or edges in specific lists.

Other approaches attempt to exchange the used data structures during run-time. *Just-in-Time* data structures (JitDS) (DeWael et al. 2015) is an extension of the Java language enabling the combination of multiple representations for a single data structure. For each instance, swap rules can be defined by an expert programmer to declare when and how to switch between representations. While this approach is powerful, it relies on the programmer’s intuition and foresight to define such rules. *Chameleon* (Shacham et al. 2009) provides a framework for run-time profiling without the need to adapt the program. In case the program uses data structure wrappers provided by the framework, data structures can be replaced during runtime which comes at the high cost of performing a separate monitoring of all data structures. Based on fixed rules for exchanging data structures as well, *CoCo* (Xu 2013) requires the programmer to use wrappers provided by the framework in order to optimize the selected data structures during run-time. With their use of pre-defined rules that do not adapt to the current properties of the graph and read accesses of the analysis, both approaches are not suited for the analysis of dynamic graphs.

In previous work (Schiller et al. 2015), we presented a compile-time approach for optimized data structure selection in the context of dynamic graph analysis. We benchmarked five data structures as potential candidates and evaluated our approach for the computation of three graph metrics. In this article, we extend this work by benchmarking a total of seven data structures, creating actual estimation functions via curve fitting, and evaluating the impact on a total of seven graph metrics. Furthermore, we propose and evaluate a run-time approach for the selection of optimal data structures during the execution of an application to handle highly dynamic workloads.

The remainder of this article is structured as follows: We introduce our terminology in Section “Terminology and notation”. In Section “Compile-time selection of efficient data structures”, we describe our compile-time approach, discuss benchmarking and profiling results, and evaluate its performance benefits. We outline and evaluate our run-time approach in Section “Run-time selection of efficient data structures” and summarize our work in Section “Summary, conclusion, and outlook”.

## Terminology and notation

In this Section, we introduce our terminology and notations for graphs, dynamic graphs, and their analysis. We introduce the different lists for representing graphs in memory as well as the operations required to adapt them over time and access them for analysis. Finally, we define the problem of selecting the best data structures for these lists.

### Graphs and adjacency lists

A *graph* *G*=(*V*,*E*) consists of a vertex set *V*={*v*_1_,*v*_2_,… } and an edge set *E*. In undirected graphs, edges are unordered pairs of vertices and ordered pairs in directed graphs. The *adjacency list* of a vertex in an undirected graph is then defined as *a* *d* *j*(*v*):={{*v*,*w*}∈*E*}. For directed graphs, *incoming* and *outgoing adjacency lists* are defined by *i* *n*(*v*):={(*w*,*v*)∈*E*} and *o* *u* *t*(*v*):={(*v*,*w*)∈*E*}. In addition, the vertices with bidirectional connections are commonly stored in the *neighborhood list*, i.e., *n*(*v*):={*w*∈*V*:(*w*,*v*)∈*i* *n*(*v*)∧(*v*,*w*)∈*o* *u* *t*(*v*)}.

### Dynamic graphs

As a *dynamic graph*, we consider a graph whose vertex and edge sets change over time. Each change is represented by an update of *V* or *E* that adds or removes an element. Applying any of these updates *a* *d* *d*(*v*), *r* *e* *m*(*v*), *a* *d* *d*(*e*), and *r* *e* *m*(*e*) implies the modification of *V*, *E*, and adjacency lists.

We consider a dynamic graph at an initial state *G*_0_=(*V*_0_,*E*_0_) and its development over time: *G*_0_,*G*_1_,*G*_2_,…. The transition between two states *G*_*i*_ and *G*_*i*+1_ of the graph can then be described by a set of updates we refer to as a batch *B*_*i*+1_. Then, the complete transition of a dynamic graph over time can be understood as the consecutive application of batches to it: $G_{0} \stackrel {B_{1}}{\longrightarrow } G_{1} \stackrel {B_{2}}{\longrightarrow } G_{2} \stackrel {B_{3}}{\longrightarrow } \dots $.

### Analysis of dynamic graphs

Analyzing a dynamic graph means to determine its topological properties at certain states, e.g., for *G*_0_,*G*_1_,*G*_2_,…. Examples of such topological metrics are the degree distribution (*DD*), connected components (*C*), assortativity (*ASS*), clustering coefficient (*CC*), rich-club connectivity (*RCC*), all-pairs-shortest paths (*SP*), and betweenness centrality (*BC*).

### Representing a dynamic graph in memory

For directed and undirected graphs, different lists are required to represent the graph and all adjacencies in memory. For both types, the set of all vertices *V* and the set of all edges *E* must be stored. For each vertex of an undirected graph, the list of all adjacent edges *adj* must be represented. In the case of directed graphs, separate lists of incoming and outgoing edges (*in* and *out*) as well as neighboring vertices (*n*) must be maintained. Hence, there is a total of 6 different lists which we denote as ${\mathcal {L}} := \{V, E, adj, in, out, n\}$. Each list stores either edges (*e*) or vertices (*v*), denoted as ${\mathcal {T}} := \{v, e\}$. We refer to this element type stored in a list by $t: {\mathcal {L}} \rightarrow {\mathcal {T}}$ with *t*(*V*)=*t*(*n*):=*v* and *t*(*E*)=*t*(*i* *n*)=*t*(*o* *u* *t*)=*t*(*a* *d* *j*):=*e*.

Each list must provide operations to modify it and retrieve certain information. To create and maintain a list, it must provide means to be initialized (*init*), add elements to it (*add*), and remove existing elements (*rem*). It must provide operations to fetch a specific element using a unique identifier (*get*) or iterate over all elements (*iter*). Often, it is also necessary to retrieve a random element from a list (*rand*), determine its cardinality (*size*), or determine if a specified element is contained in the list (*cont*).

The execution of *add*, *rem*, and *get* can be successful or fail depending on the current state of the list. Likewise, the execution of *cont* can return true or false. For example, adding vertex *v* to *V* fails in case it already exists while the removal of *e* from *E* is successful in case the edge exists. Similarly, the result of a *contains* operation can be *true* or *false*, also considered as success or failure. Depending on the data structure used to implement a list for storing elements of a specific type, the runtime can differ significantly when an operation fails compared to a successful execution. We do not need to make this distinction for the other operations: *size* and *iter* can not fail and *rand* returns *null* in case the list is empty.

Therefore, we distinguish between successful (*s*) and failed (*f*) execution of *add*, *rem*, *get*, and *cont* and consider a set ${\mathcal {O}}$ of 12 different operations: $o \in {\mathcal {O}} := \{init, add_{s}, add_{f}, rem_{s}, rem_{f}, get_{s}, get_{f}, iter, rand, size, cont_{s}, cont_{f} \}$.

### Problem definition

In this article, we consider the problem of finding the most efficient data structures for representing a dynamic graph during analysis in memory. Assume ${\mathcal {D}}$ to be a set of data structures that implement all required operations. Then, we must find the most efficient configuration *cfg* which maps each list to a data structure: $cfg: {\mathcal {L}} \rightarrow {\mathcal {D}}$. For undirected graphs, this means to select data structures for *V*, *E*, and *adj* while directed graphs require data structures for *in*, *out*, and *n* in addition to *V* and *E*. In the following, we focus on undirected graphs since all results can be transferred to directed graphs.

## Compile-time selection of efficient data structures

In this Section, we describe a compile-time approach for the selection of efficient data structures for the analysis of dynamic graphs. Afterwards, we discuss benchmarking results for different data structures and give examples. Then, we present results of operation counts obtained during profiling for the computation of graph metrics and the adaptation of a dynamic graph. Finally, we evaluate our approach on two real-world datasets and summarize our results.

### Compile-time approach

Our approach for optimizing the data structure selection for dynamic graph analysis is based on the assumption that workload and characteristics of the dynamic graph do not change drastically over time. We refer to such a workload as constant and call a workload non-constant in case access patterns or list sizes change significantly over time. In this case, we can estimate the workload for the complete analysis based on the first batches and determine the best configuration.

To understand and estimate the performance of data structures when executing specific operations, we benchmark them beforehand. This preparation phase must be executed only once for a platform where the application should be executed.

**Fig. 2:**
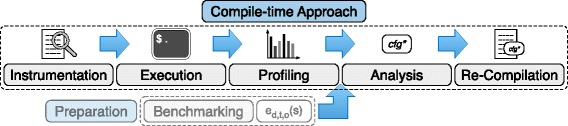
Compile-time approach for the selection of efficient data structures

An overview of our compile-time approach is given in Fig. 2 and it consists of five components: First, a given application is instrumented to enable profiling. Second, it is executed for some batches to record access statistics for all lists. Third, these access statistics are aggregated by the profiling component. Fourth, these statistics are analyzed using the runtime estimations obtained during benchmarking to recommend the most efficient configuration. Fifth and finally, the program is re-compiled to use the recommended configuration.

#### Benchmarking

The runtime of executing an operation $o \in {\mathcal {O}}$ on a list $l \in {\mathcal {L}}$ depends on the element type $t(l) \in {\mathcal {T}}$, the data structure $d \in {\mathcal {D}}$ used to implement the list, and its size $s_{l} \in \mathbb {N}^{+}$. To estimate this runtime, we perform measurements for data structures and element types with all operations and list sizes *s*∈[1,*s*_*max*_]. As a result, we obtain a set of measurements for each list size *s*: $m_{d,t,o}: [1,s_{max}] \rightarrow \mathbb {R}^{k}$.

To obtain an estimation function *e*_*d*,*t*,*o*_ from the runtime measurements *m*_*d*,*t*,*o*_, we fit the following functions using the *nonlinear least-squares (NLLS) Marquardt-Levenberg* algorithm provided by *gnuplot* ^1^:

- *f*_1_(*x*)=*a*+*b*·*x*+*c*·*x* ^2^
- *f*_2_(*x*)=*a*+*b*·*l* *o* *g*(*x*)

We chose these functions to reflect the complexity classes *O*(1), *O*(*s*), *O*(*s* ^2^), and *O*(*l* *o* *g*(*s*)) of the operations on different data structures. We fit *f*_1_ and *f*_2_ via median value and standard deviation of the data points in *m*_*d*,*t*,*o*_ and select the function with the smallest error as *e*_*d*,*t*,*o*_.

#### Instrumentation, execution, and profiling

Two actions are performed during the analysis of a dynamic graph: graph modification and metric computation. Graph modification means that the in-memory representation is changed to reflect the updates that occur in the graph over time, i.e., *add* and *rem*. For the computation of metrics, read operations like *iter*, *size*, and *contains* are executed on certain lists depending on metrics and algorithms.

In the first part of our approach, we instrument a given application such that these accesses to data structures can be recorded. Then we execute the instrumented application for some batches and aggregate the recorded access statistics for each list *l* and *o* as $c_{l}: {\mathcal {O}} \rightarrow \mathbb {N}$. We refer to *c*_*l*_ as operation counts. In addition, we record the average size of all instances of list *l* as *s*_*l*_. For example, *c*_*V*_(*a* *d* *d*) records how many elements have been added to *V* and *s*_*adj*_ denotes the average size of all adjacency lists *adj*.

#### Analysis and re-compilation

The analysis component takes as input operation counts *c*_*l*_ and average size *s*_*l*_ for all lists *l* generated during profiling. From that, we estimate the runtime of any data structure *d* as $\sum _{o \in {\mathcal {O}}} c_{l}(o) \cdot e_{d,t(l),o}(s_{l}).$ Then, the most efficient data structure $d^{*} \in {\mathcal {D}}$ for executing *c*_*l*_ for *s*_*l*_ can be estimated by

$$ d^{*}(c_{l}, s_{l}) = \arg \min_{d \in {\mathcal{D}}} \sum_{o \in {\mathcal{O}}} c_{l}(o) \cdot e_{d,t(l),o}(s_{l}). $$

Hence, the most efficient configuration for all lists *l* can be estimated as

$$ cfg^{*}(l) := d^{*}(c_{l}, s_{l}), \; l \in {\mathcal{L}}. $$

As a result, the analysis components returns the configuration *c* *f* *g* ^∗^ which was estimated to be the most efficient for executing the operation counts for the given list sizes. Finally, we re-compile the application to use *c* *f* *g* ^∗^.

### Benchmarking results

We performed a measurement study of Java data structures to obtain *m*_*d*,*v*,*o*_(*s*) and *m*_*d*,*e*,*o*_(*s*) for sizes *s*∈[1,10^5^], and seven data structures that provide the required operations: *Array* (A), *ArrayList* (AL), *HashArrayList* (HAL), *HashMap* (HM), *HashSet* (HS), *HashTable* (HT), and *LinkedList* (LL), i.e., ${\mathcal {D}} = \{A, AL, HAL, HM, HS, HT, LL\}$. *HashArrayList* is an implementation that stores all elements simultaneously in a HashSet and an ArrayList to take advantage of their respective performance for different operations as proposed by Xu (2013). For the other data structures, we used the default Java implementations.

All measurements are executed on an *HP ProLiant DL585 G7* server running a Debian operating system with 64 2.6GHz *AMD OpteronTM 6282SE* processors. We guaranteed that no more than 60 processes were running during the evaluation executed using a 64-bit JVM version 1.7. Our implementation of the benchmarking phase is available as an open-source repository^2^.

**Fig. 3:**
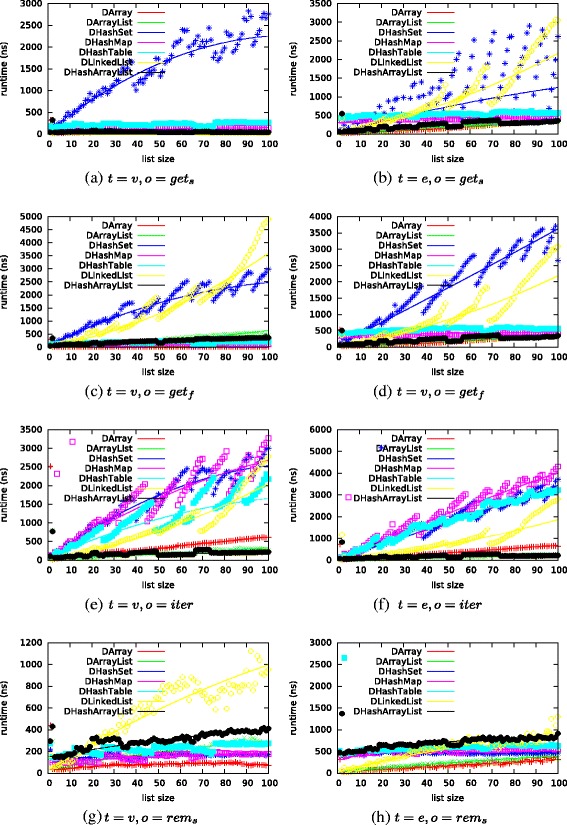
Selected runtime estimations (fitted functions *e*_*d*,*t*,*o*_ with median of 50 measurements *m*_*d*,*t*,*o*_) for list sizes *s*∈[1,100]

We used implementations of vertices and edges and repeated all measurements 50 times. A vertex *v* is identified by a unique index *i* *d*(*v*). An edge *e*=(*v*,*w*) is identified by a 32-bit (*int*) hash computed from the indexes of the connected vertices, i.e., *h*(*e*):=(*i* *d*(*v*)+*i* *d*(*w*)·2^16^) *m* *o* *d* 32. Selected results for *m*_*d*,*e*,*o*_ and *e*_*d*,*e*,*o*_ with *s*∈[1,100] are given in Fig. 3. Measurements for all operations and list sizes can be found in the technical report^3^.

As examples, we list the estimation functions for *g* *e* *t*_*s*_ and *g* *e* *t*_*f*_ in Table 1.

**Table 1:** Estimation functions of *g* *e* *t*_*s*_ and *g* *e* *t*_*f*_ depending on data structure and element type

*t*	*d*	$e_{d,t,get_{s}}(x)$	$e_{d,t,get_{f}}(x)$
*v*	A	23.74+0.91·*x*−0.01·*x* ^2^	16.72+0.15·*x*−0.00·*x* ^2^
	AL	24.49+1.41·*x*−0.01·*x* ^2^	41.09+1.82·*x*+0.04·*x* ^2^
	HAL	47.58+0.18·*x*−0.00·*x* ^2^	60.36+3.23·*x*−0.00·*x* ^2^
	HM	73.57+0.93·*x*−0.00·*x* ^2^	57.48+15.46·*l* *o* *g*(*x*)
	HS	56.20+40.23·*x*−0.18·*x* ^2^	54.05+40.99·*x*−0.17·*x* ^2^
	HT	153.87+18.14·*l* *o* *g*(*x*)	98.70+19.96·*l* *o* *g*(*x*)
	LL	39.80+0.24·*x*−0.00·*x* ^2^	26.28+14.04·*x*+0.22·*x* ^2^
*e*	A	22.92+1.88·*x*+0.02·*x* ^2^	27.78+1.51·*x*+0.02·*x* ^2^
	AL	23.49+3.65·*x*−0.00·*x* ^2^	29.81+3.63·*x*−0.00·*x* ^2^
	HAL	51.42+5.26·*x*−0.02·*x* ^2^	53.08+4.77·*x*−0.02·*x* ^2^
	HM	371.51+1.38·*x*−0.00·*x* ^2^	357.04+1.44·*x*−0.00·*x* ^2^
	HS	33.45+15.87·*x*−0.04·*x* ^2^	69.20+34.08·*x*+0.01·*x* ^2^
	HT	442.95+2.09·*x*−0.01·*x* ^2^	407.83+5.01·*x*−0.04·*x* ^2^
	LL	31.36+11.18·*x*+0.10·*x* ^2^	35.44+10.59·*x*+0.11·*x* ^2^

The fastest data structure for each operation and list sizes between 10 and 100,000 based on our estimation functions is given in Table 2. The runtime for certain operations differs greatly for data structures and list sizes. For example, *Array* is the fastest data structure for testing the existence of an edge for small list sizes (*s*=10) while *HashSet* or *HashArrayList* are the better choice for larger lists. Adding an edge to a list of sizes 10 or 100 is fastest for *ArrayList* while hash-based data structures should be preferred for larger lists.

**Table 2:** Fastest data structure according to our estimation for different list sizes

*o*	*v*					*e*				
	10^1^	10^2^	10^3^	10^4^	10^5^	10^1^	10^2^	10^3^	10^4^	10^5^
*init*	LL	LL	LL	LL	LL	LL	LL	LL	LL	LL
*a* *d* *d*_*s*_	AL	HS	HAL	HAL	HS	AL	AL	HS	HT	HT
*a* *d* *d*_*f*_	A	A	A	HS	A	A	HS	HS	HS	HS
*r* *e* *m*_*s*_	A	A	A	A	A	A	A	HS	HM	HM
*r* *e* *m*_*f*_	A	A	A	A	A	AL	HS	HS	HS	HM
*g* *e* *t*_*s*_	A	LL	A	A	LL	A	HAL	LL	HM	HT
*g* *e* *t*_*f*_	A	A	A	A	A	A	HAL	LL	HM	HM
*iter*	AL	HAL	HAL	HAL	LL	AL	HAL	LL	LL	A
*rand*	A	HAL	A	A	A	AL	HAL	A	A	HAL
*size*	A	LL	A	A	A	A	A	A	A	HAL
*c* *o* *n* *t*_*s*_	A	A	A	A	LL	A	HS	HS	HAL	HS
*c* *o* *n* *t*_*f*_	A	A	A	A	HS	A	HS	LL	HM	HS

*A* Array, *AL* ArrayList, *HAL* HashArrayList *HM* HashMap, *HS* HashSet, *HT* HashTable, *LL* LinkedList

For storing vertices, *Array* and *HashArrayList* appear to be the fastest data structures overall (cf. Table 2). They perform best for most operations and list sizes.

When storing edges, *Array* and *ArrayList* are only fast for small lists of size 10. As the lists grow, the fastest data structure depends on the respective operation and even changes again the more the lists grow (cf. Table 2). For example, *HashSet* and *HashTable* perform best when executing *a* *d* *d*_*s*_ on lists of size ≥ 1,000 while *ArrayList* is fastest for lists of size 10 and 100.

The reason for the difference in performance when storing vertices or edges lies in the identification of elements. Vertices are identified by a unique identifier which can simply be used as the index of *Array*, *ArrayList*, or *HashArrayList*. Therefore, performing *contains* or *get* operations translates to a simple lookup at a deterministic location in memory. In contrast, hash-based data structures perform the overhead of looking up this identifier in the corresponding hash table and potentially determining its location in memory. Edges are identified by a hash computed from the two unique indexes of the adjacent vertices. Their lookup in an array-based data structure is time consuming since the complete list has to be scanned. Representing all possible indexes of an edge list in an array-based data structure would require each list to map all possible hash values, and hence always be of size 2^3^2 which is infeasible. While the lookup in array-based data structures is still faster for small lists, hash-based data structures are faster for larger lists as they only need to check for the respective hash in their hash table.

From these results, we assume that array-based data structures should be recommended for storing vertices. Similarly, we see that for storing small edge lists, array-based data structures should be recommended as well. For larger edge lists with more than 100 elements, there is not a single data structure which appears best. Hash-based data structure perform better than *Array* and *ArrayList* but which one depends on the combination and count of the performed operations.

### Profiling results

We instrumented the graph component of *DNA (Dynamic Network Analyzer)* ^4^, a framework for the analysis of dynamic graphs (Schiller and Strufe 2013), to record *c*_*l*_ and *s*_*l*_ for all lists $l \in {\mathcal L}$ during graph modification and metric computation using *AspectJ* (Kiczales et al. 2001). In the following, we present such results generated using the profiling component. With these operation counts and average list sizes, we can perform an analysis to estimate the most efficient configuration.

**Fig. 4:**
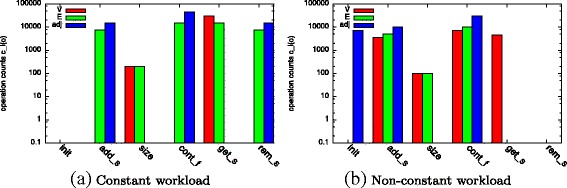
Operation counts for graph modification (*o*∈{*i* *n* *i* *t*,*a* *d* *d*_*s*_,*s* *i* *z* *e*,*c* *o* *n* *t*_*f*_,*g* *e* *t*_*s*_,*r* *e* *m*_*s*_})

First, we compare *c*_*l*_ for two different workload types of dynamic graphs: *constant* and *non-constant workload*. We refer to a workload as *constant* in case the list sizes and operation counts do not change significantly over time. In the example shown in Fig. 4 a, batches only consist of a similar amount of edge removals and additions. Such a workload is characterized by an equal number of additions and removals to *E* and *adj* without additions to *V*. We consider a workload as *non-constant* in case the list sizes or operation counts change over time. Such a workload is produced when growing a graph, i.e., adding new vertices and further interconnecting them (cf. Fig. 4 b). This workload is reflected by add operations on *V*, *E*, and *adj* but not a single removal.

**Fig. 5:**
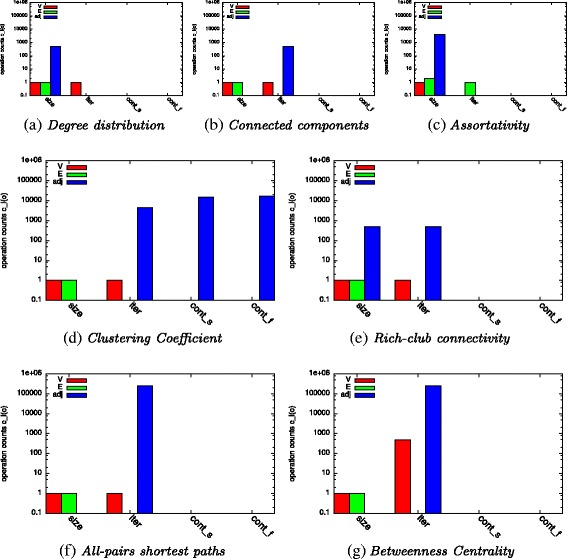
Operation counts for metric computation (*o*∈{*s* *i* *z* *e*,*i* *t* *e* *r*,*c* *o* *n* *t*_*s*_,*c* *o* *n* *t*_*f*_})

Second, we observe *c*_*l*_ during the computation of seven metrics on an instance of a dynamic graph: degree distribution, connected components, assortativity, clustering coefficient, rich-club connectivity, all-pairs shortest paths, and betweenness centrality. We selected these metrics to cover all operations and their combinations commonly found in graph analysis^5^. To compute the degree distribution of a graph, an algorithm iterates once over *V* and determines the degree of each vertex using the size operation of its adjacency list *adj* (cf. Fig. 5 a). Similar operation counts can be observed for the rich-club connectivity (cf. Fig. 5 e) with the difference that the iteration is performed over *E* instead of *V*. To determine the connected components of a graph, a breadth-first search is performed by iterating over *V* and the adjacency lists *adj* (cf. Fig. 5 b). All-pairs-shortest paths and betweenness centrality are computed by performing similar operations from every vertex resulting in a higher count (cf. Fig. 5 f and g). Computing the clustering coefficient of a graph implies an iteration over all vertices and iterations over all adjacency lists *adj* (cf. Fig. 5 d). On these adjacency lists, contains operations are executed to check which neighbors of a vertex are connected to each other. Some of these operations fail, others are successful.

During the profiling phase, executed for each program at the beginning of our compile-time approach, the counts for graph modification as well as metric computation are recorded and used as basis for the recommendation.

### Evaluation

Now, we evaluate our compile-time approach on the analysis of two real-world dynamic graphs: one that produces a constant workload (*MD*) and a second one that generates a non-constant workload (*FB*). Our analysis scripts for performing the evaluation are available as an open-source repository^6^.

#### Datasets

**Fig. 6:**
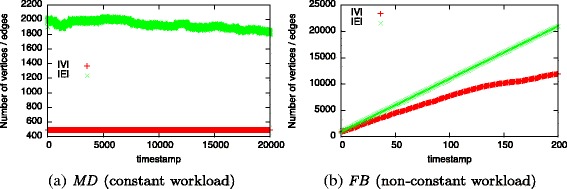
Dataset statistics (development of |*V*| and |*E*| over time)

*MD* is the dynamic graph obtained from a molecular dynamics simulation of an enzyme, the para Nitro Butyrate Esterase-13 (Schiller et al. 2015). The initial graph consists of 491 vertices representing the residues of the enzyme and 1,904 edges. Edges exists between two vertices in case their Euclidean distance is shorter than $7\dot {A}$. During the simulation, a total of 20,000 snapshots were taken. On average, each batch consists of 70 edge additions and 70 edge removals resulting in a constant workload (cf. Fig. 6 a).

The *FB* dataset is a friendship graph of Facebook taken from KONECT, the Koblenz Network Collection (Kunegis 2013). It represents users and their friendship relations as a list of edges sorted by the timestamp they appeared. We take the initial graph consisting of the first 1,000 edges and 898 vertices. With each batch, the next 100 edges and corresponding vertices are added creating a non-constant workload. After 200 batches, the graph consists of 11,941 vertices and 21,000 edges (cf. Fig. 6 b).

For both datasets, we create the initial graph and apply the first 20 batches. After the application of each batch one of the following metrics was computed: *DD*, *C*, *RCC*, *ASS*, *SP*, *CC*, or *BC*. Based on the operation counts *c*_*l*_ of the 20 batch applications and metric computations, we determine the recommended data structures for *V*, *E*, and *adj*.

Then, we perform the same computation with the recommended data structures, as well as configurations where *V*, *E*, and *adj* are all using *Array*, *ArrayList*, *HashArrayList*, *HashMap*, *HashSet*, *HashTable*, or *LinkedList*, referred to as *basic configurations*. In total, we compute the properties of *MD* for all 20,000 states and the properties of *FB* for 201 states. For comparison, we compute the runtime of all seven configurations relative the configurations recommended by out approach. All results presented here are the median speedup of 50 repetitions.

#### Constant workload

For *MD*, our approach recommended the use of *HashMap* for *E* for all metrics (cf. Table 3). It recommended to use either *Array* or *ArrayList* for *adj* and *Array* or *HashArrayList* for *V*. Since the dataset creates a constant workload, we expect that our recommendation is applicable and therefore well-suited for the analysis of the complete dataset.

**Table 3:** Recommendations for *V*, *E*, and *adj* depending on workload and computed metric

Metric	Constant workload (*MD*)			Non-constant workload (*FB*)		
	V	*E*	*adj*	*V*	*E*	*adj*
All-pairs shortest paths	A	HM	AL	HAL	HAL	LL
Assortativity	A	HM	A	HAL	HAL	A
Betweenness centrality	HAL	HM	AL	LL	HAL	LL
Clustering coefficient	A	HM	A	HAL	HAL	AL
Degree distribution	A	HM	A	HAL	HAL	AL
Rich-club connectivity	A	HM	AL	HAL	HAL	AL
Connected components	A	HM	AL	HAL	HAL	AL

**Fig. 7:**
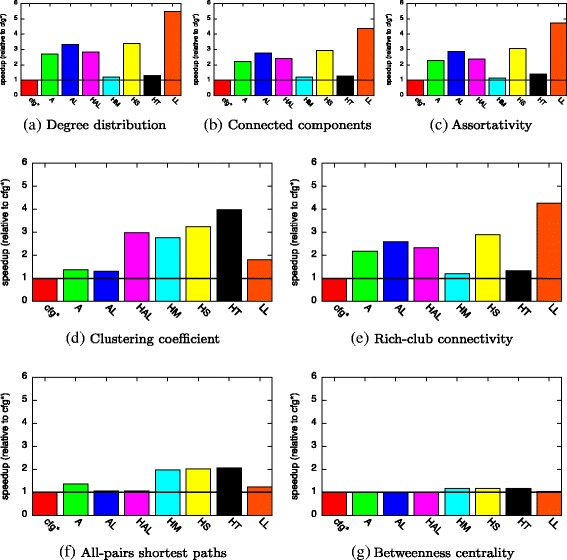
Speedup of compile-time approach (for analysis of constant workload (*MD*))

The relative speedup of our recommended configurations over all seven basic configurations is given in Fig. 7. Our recommended data structures achieve a speedup up to 5.4× and always outperform the basic configurations. The relative performance is very similar when computing degree distribution, connected components, and assortativity. This is most probably because these three metrics have a similar access pattern to the graph (cf. Fig. 5 a, b, and c). For the other metrics (*CC*, *RCC*, *SP*, and *BC*), the relative speedup greatly differs with no basic configuration outperforming the others.

#### Non-constant workload

After profiling for the first 20 batches of *FB*, our approach recommended the use of *HashArrayList* for representing *E* for all metrics. With a single exception, the same data structure was recommended for *V* while the use of either *Array*, *ArrayList*, or *LinkedList* was proposed for *adj*. We consider this workload to be non-constant because the sizes of *V* and *E* increase with each batch. We expect that this significant change in list sizes renders the initial profiling meaningless for the far longer running analyses of all 200 batches. Based on the profiling during the first twenty batches, we assume a total number of 1,000+20·100=3,000 edges as input of our analysis. But after 200 batches, *E* grows to a total of 21,000 elements, 7× more than the list size we assume based on our initial profiling. Therefore, we expect that the recommendations generated by our approach are not always the best choice throughout an analysis and can be outperformed by the other configurations.

**Fig. 8:**
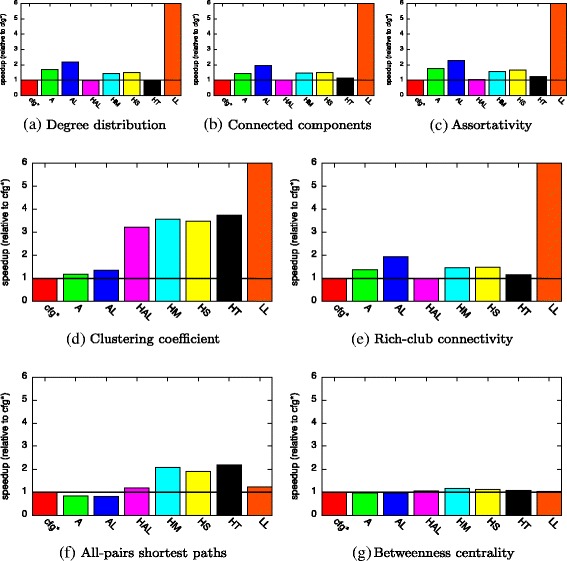
Speedup of compile-time approach (for analysis of non-constant workload (*FB*))

The relative speedup for the analysis of *FB* for all metrics is shown in Fig. 8. Note that the speedup for *LinkedList* lies between 7.5 and 245 for computing *DD*, *C*, *ASS*, and *CC* and is truncated in these plots. As for the constant workload, the relative speedups for computing degree distribution, connected components, and assortativity are similar. For all metrics, there is at least one standard configuration that closely matches the runtime of the recommended data structures. When computing all-pairs shortest paths, the standard configurations with *Array* and *ArrayList* outperform our recommendations with only 80 % of the total runtime.

### Summary of the compile-time approach

The fact that our recommended configurations outperform all other tested combinations for *MD* suggests that our estimation of the actual runtime based on *e*_*d*,*t*,*o*_ is accurate and the recommendation valid for all subsequent batches. We have shown that our compile-time approach achieves speedups over basic configurations in case of a constant workload. These recommendations are based on a short profiling phase and the results independent of the duration of the analysis afterwards.

In contrast, our evaluation has shown that our compile-time approach is not always able to accelerate the analysis for all metrics when applying a non-constant workload (*FB*). We assume that this is because of the increase of list sizes over the complete analysis period which also affects the operation counts.

Hence, we conclude that our compile-time approach is well suited for constant but not for non-constant workloads. Therefore, we propose a run-time approach that analyzes the workload during the execution of an application and exchanges data structures accordingly to account for changes in list sizes and operation counts over time.

## Run-time selection of efficient data structures

In this Section, we present a run-time approach for the selection of efficient data structures for the analysis of dynamic graphs. Then, we perform a performance analysis using an artificial workload. Finally, we summarize the insights gained from the analysis.

### Run-time approach

For our run-time approach, we assume that the workload (i.e., list sizes or operation counts) of an application changes drastically over time. In such a case, there is not a single data structure configuration which performs best throughout the complete execution and it would be necessary to continually change the data structures during execution for optimal performance. Based on this assumption, we propose an approach to monitor the list sizes and operation counts at run-time, use that information to make regular recommendations for the best configuration for the current workload, and finally exchange the data structures used to represent the dynamic graph in memory.

**Fig. 9:**
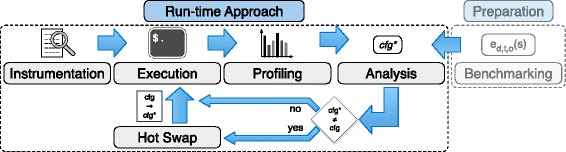
Run-time approach for the selection of efficient data structures

Our approach for the run-time optimization of graph data structures consists of the following components, shown in Fig. 9: instrumentation, execution, profiling, analysis, and hot swap.

The instrumentation adds capabilities to the program to record the access statistics and list sizes during execution and perform a hot swap of data structures if required. Like in our compile-time approach, the profiling component regularly generates operation counts and average list sizes. The analysis component takes these statistics as well as the cost functions generated during the benchmarking phase as input to recommend a data structure configuration. In case this recommendation differs from the currently used configuration, the hot swap component replaces the lists in memory with new instances of the recommended data structure. Afterwards, the execution of the program is continued.

**Hot Swap** In our compile-time approach, the recommended data structures are assigned to the respective lists and the program is re-compiled. In the run-time approach, these changes must be applied during the execution of the program. In case a new recommendation appears more efficient than the current one, we pause the execution and exchange the current data structures for the recommended ones. To exchange the data structure we create new instances of the recommended data structure and fill them with the elements representing the current state of the graph. Afterwards, we update all references that point to the respective list.

### Performance analysis

To analyz the performance of our run-time approach, we generated an artificial workload where the operations executed on *V* and *E* as well as their sizes change over time to investigate how our approach performs compared to basic configuration for highly dynamic scenarios. We execute this workload for each of the 7 basic data structure configurations we used before and for our run-time approach. The run-time approach always begins execution using *Array* as the data structure for all lists. For each execution, we measure the runtime for processing the workload as well as the overhead of recommending data structures and exchanging them.

**Workload and execution** To understand the characteristics of the performance in detail, we designed a synthetic workload. It consists of 4 separate steps, each of which is applied first to *V* and then *E*, resulting in a total of 8 different operations on the data structures:

1. *cont:V*, *cont:E* - 100*k* contains operations of random elements
2. *get:V*, *get:E* - 100*k* get operations of random elements
3. *iter:V*, *iter:E* - 10*k* iterations over all elements
4. *add:V*, *add:E* - 1*k* additions of new elements

Each of these individual operations is performed 10 times before moving on to the next, forming a round consisting of 80 operations. We execute 4 such rounds, leading to a total of 320 separate operations.

**Fig. 10:**
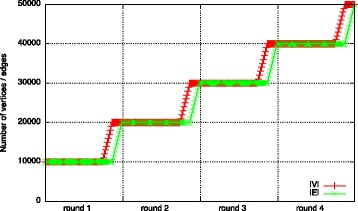
List sizes (development of |*V*| and |*E*| during application of the artificial workload)

We start such an execution with a random graph consisting of 10*k* vertices and edges. We then apply *add:V* and *add:E* 10 times at the end of each round, leading to a final list size of 50*k* elements once the workload has finished executing (cf. Fig. 10).

All runtimes shown in the following are the average of 50 repetitions.

**Fig. 11:**
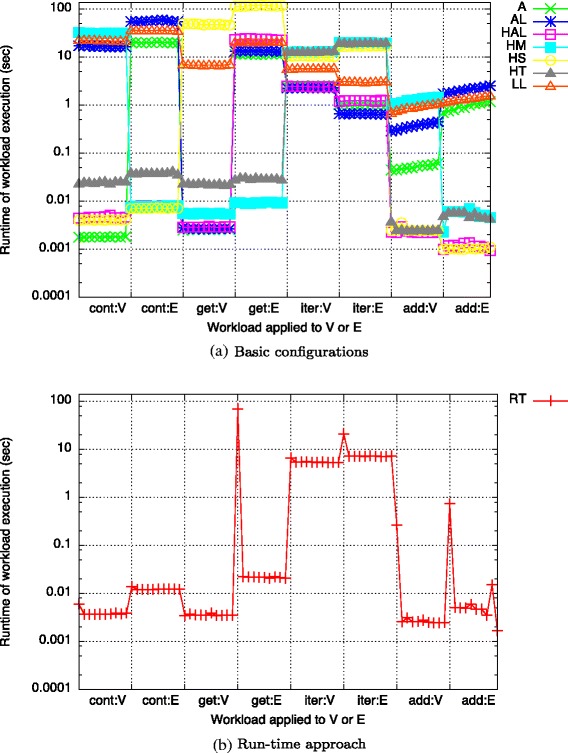
Workload runtimes (execution of artificial (non-constant) workload, round 3)

**Basic configurations** The runtimes for executing a single round of the workload using the seven basic configurations are shown in Fig. 11 a.

As the sizes of *V* and *E* do not change during the execution of *cont*, *get*, and *iter*, their runtimes only depend on the data structure used but remain similar for all repetitions. In contrast, each application of *add:V* and *add:E* increases the respective list size by 1*k* leading to an increase in their runtime with each repetition.

As indicated by our benchmarks, array-based data structures (*Array*, *ArrayList*, *HashArrayList*) are most efficient for the execution of *cont:V*, *get:V*, and *iter:V*. For *add:V*, hash-based data structures (*HashArrayList*, *HashSet*, *HashTable*) perform best.

For operations executed on *E*, the results are more diverse: While *HashArrayList*, *HashMap*, and *HashSet* are the best choices when executing *cont:E*, *HashMap* is the fastest data structure for obtaining elements (*get:E*). When executing *iter:E*, *ArrayList* performs best. When adding elements, all hash-based data structures (*HashArrayList*, *HashMap*, *HashSet*, *HashTable*) outperform the others.

*HashArrayList* always performs well when either *HashSet* or *ArrayList* do so. This is expected because *HashArrayList* takes advantage of their respective benefits to execute these operations and shows the usefulness of this combined data structure.

**Run-time approach** The best data structure for the execution of an operation depends on the element type and its size. Therefore, the data structures recommended by the analysis component of our run-time approach should change accordingly as the artificial workload is executed. These recommendations, depending on operation, element type, and size are shown in Table 4.

**Table 4:** Recommended data structures (for workload and set size, underlined: swap required)

list size	cont:V	get:V	iter:V	add:V	cont:E	get:E	iter:E	add:E
10*k*	*A*	*A*	AL	HS	HAL	HM	AL	HS
20*k*	A	*A*	AL	HS	*HS*	HM	AL	HS, HM
30*k*	A	*A*	AL	HS	HS	HM	A	HM
40*k*	A	*A*	AL	HS	HS	HM	A	HM

Our approach correctly recommends the data structure which ran the fastest during the execution using the basic configurations (cf. Fig. 11 a): For all investigated list sizes, *Array* is recommended for the execution of *cont:V* and *get:V*. When executing *get:V*, *ArrayList* is proposed and *HashSet* for adding vertices (*add:V*). When obtaining elements from *E* (*get:E*), *HashMap* is recommended for all sizes. For the execution of *cont:E*, *HashArrayList* is recommended for list sizes below 20*k* while *HashSet* is selected for larger ones. Similarly, *Array* is recommended for executing *iter:E* on lists with 30*k* and more elements but *ArrayList* for smaller ones. When executing *add:E*, the recommendation changes during the second round: *HashSet* is recommended for |*E*|≤21*k* and *HashMap* for larger ones.

The runtimes of our run-time approach (denoted as *RT*) for executing a single round of this workload are shown in Fig. 11 b. Our approach achieves runtimes consistent with the expectation of following our recommendation of the fastest basic configuration (cf. Fig. 11 a). The only anomaly introduced in the run-time approach are spikes that can occur on the first execution of each operation batch. The reason for this behavior is that we have to execute a new operation at least once on the old data structure before we can recognize that swapping the data structure would be beneficial. For example, take the execution of *get:E*: During the first execution of this operation, *E* is still stored in *HashSet*, the best choice for the previously executed *cont:E*. During this first execution, the accessed operations are recorded by the profiling component and used by the analysis component to recommend a data structure that is best suited for this new workload. Afterwards, the hot swap component replaces these data structures with the recommended ones which leads to the performance improvement for the following executions.

**Fig. 12:**
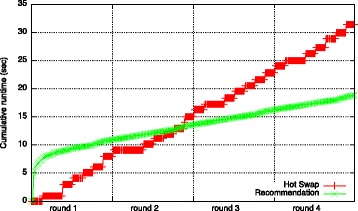
Overhead of run-time approach (consisting of hotswap and recommendation)

When using our run-time approach, overhead is produced by the recommendation of data structures and the regular execution of the hot swap component. The cumulative overhead of these two operations for all 4 runs is shown in Fig. 12. At a total execution time using our run-time approach of 821.24 *s* *e* *c*, this overhead accounts for 6,11 %. The overhead is composed of the time for recommending data structures (18.82 *s* *e* *c*, 2.29 %) and hot swap (31.38 *s* *e* *c*, 3.82 %).

**Fig. 13:**
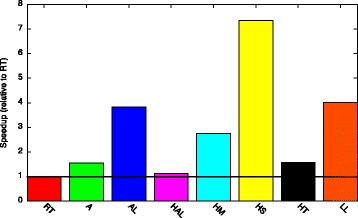
Speedup of run-time approach (for application of artificial workload, 4 rounds)

**Comparison** For the artificial workload, our approach, including its overhead, achieves a speedup over all basic configurations (cf. Fig. 13). The fastest runtime of a basic configuration is achieved by *HashArrayList* with a speed of 1.12. This is not surprising as this data structure combines the benefits of *HashSet* and *ArrayList* both of which are also recommended by our approach. The highest speedup of 7.34 is achieved in comparison to the basic configuration using *HashSet* for all lists.

### Summary of the run-time approach

We proposed a run-time approach for recommending and exchanging the data structures used to represent a dynamic graph in memory. We evaluated our approach using an artificial, regularly changing workload. Our approach outperformed basic configurations by up to 7.34×. This shows that in scenarios where the workload behavior changes over time, our approach has the potential to achieve significant performance improvements for the analysis of dynamic graphs. Some questions, however, remain open and need to be investigated in future work:

What is the best recommendation given a realistic execution history? We currently assume that any overhead is justified when making our recommendation, which is obviously not a generally valid assumption. The problem of determining whether a system has shifted its workload sufficiently that the cost of the overhead of swapping data structures is outweighed by the performance gain of a faster data structure is not trivial. This problem can be broken up into several sub-problems: How can the difference between a dynamic system changing its behavior and just making a few anomalous requests be determined? We currently assume that a realistic application of dynamic graph analysis will not erratically change its workload, but rather stay consistent to a slowly changing usage profile. We believe that this assumption is valid and supported by real world data, but the degree of consistency and the velocity of overall change varies from application to application. Determining these factors is critical in order to answer the above question and make an accurate recommendation. How much information should be taken into account when making our recommendations? This question pertains to how much of the execution history is relevant for our recommendation. On the one hand, correct processing of more information can never make the result less accurate, on the other hand taking into account too much information might make the system inflexible over time and significantly increase the overhead of our recommendation.

It may not be avoidable to use a certain degree of machine learning to make the best recommendation due to the sheer number and complexity of the involved variables.

On a lower level, closer to the implementation of data structures themselves, it should be investigated how the actual exchange of data structures can be improved. Instead of treating the swap between any two data structures over the same interfaces, more efficient ways to swap between specific data structures should be investigated.

## Summary, conclusion, and outlook

In this work, we considered the problem of finding the most efficient data structures for representing a graph for the application of dynamic graph analysis.

We proposed a compile-time approach for optimizing these data structures. As a case study, we performed a measurement study of seven data structures, fitted estimation functions from the results, implemented our approach on top of a Java-based framework for dynamic graph analysis, and evaluated it using real-world datasets. Our results show that our optimization achieves speedups of up to 5.4× over basic configurations on real-world datasets.

The data structure configuration proposed by our approach outperformed all seven default configurations for the computation of all metrics for a constant workload. For non-constant workloads, we achieved speedups in many but not all cases. Thereby, our approach is well-suited for improving the analysis of dynamic graphs with a constant workload but not capable of adapting to the drastic changes of list sizes that can occur in non-constant workloads.

To close this gap, we developed a new run-time based approach for the adaptation of graph data structures during the execution of an application. We analyzed the performance of our approach using a synthetic workload designed to capture most operations and generate a non-constant workload. In this scenario, our approach performed as expected and achieved speedups over basic configuration of up to 7.3×.

In future work, we will further investigate the benchmarking phase of our approaches to generate more appropriate cost estimation functions. In addition, we will perform an extensive parameter study to understand the different aspects of the proposed run-time approach and look for methods to determine when to use which approach.

## Endnotes

^1^ http://gnuplot.sourceforge.net

^2^ https://github.com/BenjaminSchiller/DNA.gdsMeasurements

^3^ http://bit.ly/1UT9pnX

^4^ https://github.com/BenjaminSchiller/DNA

^5^ We omitted the computation of motif frequencies used in previous work because the resulting operation counts and runtimes are very similar to those observed for the clustering coefficient.

^6^ https://github.com/BenjaminSchiller/DNA.gdsAnalysis
